# 17q12 Recurrent Deletion Syndrome in Childhood

**DOI:** 10.3390/genes16121499

**Published:** 2025-12-15

**Authors:** Giorgia Ceravolo, Salvatore Mollica, Marco Cavallaro, Ida Ceravolo, Giovanni Sica, Francesca Granata, Henry Houlden, Roberto Chimenz

**Affiliations:** 1 Department of Clinical and Experimental Medicine, University of Messina, 98122 Messina, Italy; 2 Department of Neuromuscular Disorders, Institute of Neurology, University College London, London WC1E 6BT, UK; 3 Department of Biomedical Sciences and Morphological and Functional Imaging, University of Messina, Policlinico “G. Martino”, 98100 Messina, Italy; marco88cavallaro@gmail.com (M.C.); francesca.granata@unime.it (F.G.); 4 Neonatal and Pediatric Intensive Care Unit, University Hospital “Renato Dulbecco”, 88100 Catanzaro, Italy; giovanni.sica@aourenatodulbecco.it; 5 Unit of Pediatric Nephrology, and Rheumatology with Dialysis, Department of Human Pathology in Adult and Developmental Age “Gaetano Barresi”, University of Messina, Policlinic “G. Martino”, 98125 Messina, Italy

**Keywords:** 17q12 recurrent deletion, HNF1B, paediatric nephrology, congenital anomalies of the kidney and urinary tract (CAKUT), MODY5, neurodevelopmental disorders, intrafamilial variability, chromosomal microarray

## Abstract

Background: The 17q12 recurrent deletion syndrome is a genomic disorder encompassing a 1.4 to 1.5 Mb region that includes the HNF1B gene, and it manifests with remarkable phenotypic variability. Renal anomalies, endocrine and metabolic disturbances, and neurodevelopmental or psychiatric disorders are recurrent features, although penetrance and severity differ widely between patients. Methods: We reviewed the literature on the molecular basis, clinical presentation, diagnostic approaches, and management of 17q12 deletion syndrome, and we illustrate the variability of this condition through two contrasting paediatric cases. Results: The cases concern three siblings harbouring the same familial deletion, who nevertheless exhibited striking intrafamilial variability, ranging from renal and neurodevelopmental features to multisystemic involvement. These cases exemplify both extremes of the syndrome and highlight the challenges of clinical prognostication. Conclusions: The review and cases emphasise the importance of early genetic testing in paediatric renal anomalies, the necessity of multidisciplinary surveillance even in asymptomatic individuals, and the relevance of 17q12 deletion as a model of variable expressivity in genomic medicine.

## 1. Introduction

The integration of genomic technologies into paediatric medicine has altered both diagnostic and prognostic approaches to complex disorders. Among clinically relevant copy number variants, the 17q12 recurrent deletion has become a paradigm for how structural genomic change can produce multisystem disease with highly variable expression [1,2]. The deletion encompasses a recurrent segment of approximately 1.4 to 1.5 megabases on the long arm of chromosome 17. This interval contains at least fifteen protein-coding genes, with *HNF1B* considered central to renal and metabolic manifestations [1,3]. Neighbouring genes within the interval likely broaden neurodevelopmental and psychiatric expression, which explains the wider phenotype seen in deletion carriers [4,5]. This recurrent CNV is mediated by non-allelic homologous recombination between flanking low-copy repeats.

The syndrome was first systematically characterised in 2007 in association with renal abnormalities, diabetes and epilepsy, establishing a distinct clinical entity. Subsequent reports, cohort studies and registry-based analyses expanded the recognised spectrum and placed the syndrome firmly within paediatric nephrology, endocrinology and developmental medicine [6,7,8,9]. Population prevalence is difficult to define and varies with ascertainment. National registry data suggest that diagnosed deletions are rare in the general population, whereas enrichment is expected in clinically selected cohorts (e.g., congenital kidney anomalies or early-onset diabetes) [10,11]. Asymptomatic and mildly affected carriers likely lead to under-ascertainment, so point estimates should be interpreted cautiously.

Renal involvement is frequent and can be detected prenatally or postnatally, with findings that range from subtle cortical echogenicity or microcysts to hypoplasia, dysplasia or agenesis [12,13,14]. Endocrine and metabolic complications are also reported. Maturity onset diabetes of the young type 5 linked to *HNF1B* is an important component and often presents beyond early childhood [15]. Additional features include pancreatic and hepatic involvement in a subset of patients. A major development in recent years has been the recognition of neurodevelopmental and psychiatric risks, with increased rates of autism spectrum conditions and schizophrenia in carriers and additional reports of attention deficit hyperactivity disorder, intellectual disability and motor or speech delay [16,17,18].

Both de novo and familial events occur, and expression may vary even within a single family. From a clinical perspective, the deletion exemplifies the need for multidisciplinary care. Children may come to attention through prenatal ultrasound showing echogenic kidneys, through early metabolic disturbance or through developmental assessment [19]. In each route, recognition of the deletion redirects management toward anticipatory surveillance and tailored counselling. Important questions remain regarding gene-level contributions within the interval, limits of prognostication from genotype alone and the optimal breadth and cadence of surveillance in asymptomatic children. Here we synthesise current knowledge and illustrate the range of expression through our contrasting paediatric cases.

## 2. Materials and Methods

This article combines a narrative review of the 17q12 recurrent deletion syndrome with the presentation of three illustrative paediatric cases. For the review component, a comprehensive literature search was conducted in PubMed, Scopus, and Web of Science. The search terms included “17q12 deletion”, “17q12 recurrent deletion syndrome”, “HNF1B”, “renal cysts and diabetes”, “RCAD”, “copy number variant”, and “MODY5”. Articles published in English between January 2007, when the syndrome was first characterised, and May 2025 were considered. Original case reports, case series, cohort studies, and expert reviews were included. Reports focusing exclusively on duplications of 17q12 or on other genomic regions were excluded. The bibliographies of retrieved papers were hand-searched to identify additional relevant publications. Data were extracted on genetic mechanisms, clinical presentation across organ systems, diagnostic methods, management strategies, and long-term outcomes. Given the rarity of the condition and the predominance of descriptive studies, a narrative rather than systematic synthesis was undertaken.

The cases derive from patients directly observed in our clinical practice. Clinical data were obtained from medical records, specialist consultations, and follow-up visits. Renal involvement was assessed through ultrasonography and magnetic resonance imaging, where indicated, while neurodevelopmental assessment included neurological evaluation and, where applicable, psychometric testing. Laboratory studies comprised renal function tests, electrolyte panels, glucose tolerance, and liver enzymes. Ophthalmological and neuroimaging investigations were performed in the siblings according to clinical indications. Genetic testing in all cases was performed by chromosomal microarray analysis, which identified heterozygous deletions at 17q12.

Informed consent for clinical investigations and for publication was obtained from the parents of all participating children. Ethical approval was granted by the institutional review board of our centre, and all procedures were conducted in accordance with the Declaration of Helsinki.

For the narrative synthesis, we prioritised paediatric data and, where adult-only reports were included, extracted features relevant to childhood surveillance and transition to adult care. Where cohort publications overlapped in time or recruitment centre, we retained the largest dataset and used smaller reports to clarify specific domains, such as prenatal imaging findings or neurodevelopmental outcomes. The three clinical cases were integrated as illustrative anchors to highlight variability in expression and to ground surveillance recommendations in real-world trajectories.

## 3. Clinical Spectrum of 17q12 Recurrent Deletion Syndrome

### 3.1. Genetics and Molecular Basis

**Figure 1:**
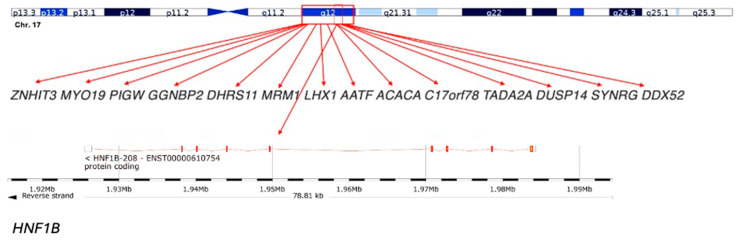
Schematic representation of the 17q12 deletion region: The 17q12 chromosomal region includes several genes, such as ZNHIT3, MYO19, PIGW, GGNBP2, DHRS11, MRM1, LHX1, AATF, ACACA, C17orf78, TADA2A, DUSP14, SYNRG, and DDX52. The HNF1B gene, which is a key gene within this region, is highlighted at the bottom. This deletion spans approximately 1.3 Mb and is associated with a variety of clinical phenotypes, including renal and neurodevelopmental disorders.

The 17q12 recurrent deletion is a structural genomic rearrangement mediated by non-allelic homologous recombination between low-copy repeats. The canonical deletion spans 1.4 to 1.5 megabases on chromosome 17q12 and encompasses at least fifteen protein-coding genes. Among these, *HNF1B* is the most extensively characterised. It encodes a transcription factor expressed during embryogenesis in the kidney, pancreas, liver, and brain, orchestrating nephrogenesis, tubular transport, pancreatic beta-cell differentiation, and hepatocellular function. Point mutations in *HNF1B* cause renal cysts and diabetes syndrome, but deletion carriers consistently display a broader phenotype, highlighting the contribution of additional genes. The canonical recurrent interval is ~1.4–1.5 Mb at 17q12 (GRCh38/hg38 approximately chr17:36,458,167–37,854,616), mediated by non-allelic homologous recombination between flanking low-copy repeats (Figure 1).

LHX1, a LIM homeobox gene, is essential for forebrain and kidney development, and its loss has been linked to hippocampal and corpus callosum abnormalities [20]. ACACA, encoding acetyl-CoA carboxylase alpha, participates in lipid metabolism and may shape metabolic features. Genes such as SYNRG and ZNHIT3 influence synaptic and neuronal pathways, providing a mechanistic basis for the strong psychiatric associations. Animal models of Hnf1b insufficiency recapitulate renal and pancreatic features; broader neurodevelopment likely reflects additive haploinsufficiency within the interval.

Population prevalence varies by ascertainment; recent estimates range from ~1:4000 to 1:50,000, with an unbiased population study suggesting ~1:6250. The syndrome appears highly penetrant; notably, there are no clear reports of unaffected carrier parents in published series.

Approximately 70 to 75% of cases arise de novo, while 25 to 30% are inherited in an autosomal dominant pattern. The clinical literature has repeatedly highlighted variability as the defining property of this syndrome. Mefford and colleagues, in their seminal description of the recurrent deletion, already noted the range of renal, metabolic, and neurological involvement. Subsequent cohorts, such as the Danish series of thirty-eight individuals, have underscored this point quantitatively, with renal anomalies present in over 80% of carriers, diabetes in about 40%, neurodevelopmental impairment in 60%, and psychiatric diagnoses in roughly one quarter, but with no consistent correlation between domains. Such figures demonstrate population-level penetrance, yet at the level of the individual family, the unpredictability is more profound [8,17]. When no family history exists, the diagnosis often follows incidental findings on prenatal or postnatal imaging. In these children, prognosis is even more opaque because there are no intrafamilial comparators. The question for clinicians is not simply whether variability exists but how to communicate that variability in a way that is honest, comprehensible, and useful for decision-making. This challenge is particularly acute in prenatal settings, where ultrasonographic detection of echogenic or cystic kidneys leads to genetic testing. The outcomes of foetal cohorts demonstrate the extremes: some children maintain stable renal function, while others progress rapidly to chronic kidney disease, and in rare cases, bilateral agenesis results in Potter sequence and neonatal death [21,22]. Families confronted with such information must make decisions about pregnancy continuation or termination in the face of irreducible uncertainty [23].

Familial transmission exemplifies the hallmark variability, as illustrated by our sibling series, in the discussion.

### 3.2. Renal Features

Renal disease represents the defining manifestation of 17q12 deletion syndrome, reported in over 80% of carriers [24]. The spectrum of congenital anomalies of the kidney and urinary tract (CAKUT) includes renal cysts (most common), renal agenesis (unilateral or bilateral), renal hypodysplasia, multicystic dysplastic kidney (MCDK), horseshoe kidney, and collecting system abnormalities such as hydronephrosis and vesicoureteral reflux [9]. Prenatal ultrasonography frequently detects echogenic kidneys, microcysts, or reduced renal size, which often prompt postnatal genetic testing. Severity ranges from subtle cortical change to lethal agenesis; severity on imaging does not reliably forecast function [25,26].

Despite the structural anomalies, renal function is unpredictable. Some carriers retain stable glomerular filtration for years, whereas others progress to chronic kidney disease (CKD) or end-stage kidney disease (ESKD) during adolescence [27]. Hypertension and proteinuria often accompany decline, but structural severity on imaging does not reliably forecast trajectory. Progression to ESKD in childhood appears uncommon; among adults, ESKD is less frequent in deletion carriers than in those with intragenic HNF1B variants.

Avoid/Use with caution: tacrolimus/mTOR inhibitors post-transplant; NSAIDs with kidney disease; hepatotoxins with liver disease; antipsychotics that promote weight gain; lithium in the setting of renal anomalies [26,27]. Histological studies from specimens provide some mechanistic insight, showing dysplastic architecture, reduced nephron endowment, and cystic dilatation consistent with disrupted nephrogenesis [28]. Yet histology is rarely available in children, leaving clinicians reliant on imaging and functional monitoring. The clinical implication is that surveillance schedules must be designed without assuming stability when function is normal and without assuming inevitable decline when imaging is abnormal. Periodic assessment of renal function, blood pressure, and proteinuria remains essential for all carriers, regardless of their initial presentation [29].

### 3.3. Endocrine and Metabolic Features

HNF1B haploinsufficiency is strongly linked to MODY5, a subtype of diabetes mellitus characterised by early-onset [30]. Insulin-requiring diabetes that responds poorly to sulfonylureas, most patients ultimately require insulin [11,31,32]. MODY5 occurs in approximately 30–50% of deletion carriers, although the onset is typically in adolescence or early adulthood. Impaired glucose tolerance or isolated fasting hyperglycaemia may precede overt diabetes [31,33]. The endocrine and metabolic dimensions of the 17q12 deletion unfold along a different temporal axis. Maturity-onset diabetes of the young type 5 is a signature feature of HNF1B disruption, yet it rarely defines the early paediatric years. In the Danish cohort, diabetes was observed in about 40%, often with onset in adolescence or early adulthood.

Electrolyte disturbances are also common. Up to ~40% of carriers develop hypomagnesaemia from renal magnesium wasting, likely reflecting distal convoluted tubule transport dysregulation downstream of HNF1B [34]. Consequences include tetany, cramps, or arrhythmias. Hyperuricaemia and early-onset gout are also observed. Pancreatic hypoplasia may compromise both endocrine and exocrine functions. Exocrine pancreatic insufficiency can cause malabsorption, steatorrhea, and fat-soluble vitamin (A, D, E, K) deficiencies [30,31]. Hypomagnesaemia, hyperuricaemia, and pancreatic exocrine insufficiency similarly tend to appear later [35,36].

This delayed emergence presents both a challenge and an opportunity. The challenge is that the absence of metabolic abnormalities at diagnosis can create a false sense of security for families. The opportunity lies in surveillance: with clear knowledge of what may arise, clinicians can establish longitudinal monitoring plans that allow early detection and timely intervention. In practice, this means scheduling regular fasting glucose or glucose tolerance tests, serum magnesium and urate levels, and liver function tests. Explaining to families that the purpose of these investigations is not to confirm an expected decline but to capture problems early reframes surveillance as a proactive safeguard rather than a marker of inevitable disease.

### 3.4. Neurodevelopmental and Psychiatric Features

One of the most transformative insights from recent studies is the recognition that the 17q12 deletion is among the most penetrant copy-number variants for autism spectrum disorder and schizophrenia. Intellectual disability, attention deficit hyperactivity disorder, epilepsy, and developmental delay are variably present, while some carriers show preserved cognition [2,16,37]. Structural neuroimaging often reveals hippocampal malformations, ventricular dilatation, or corpus callosum anomalies, which correlate with developmental outcomes. Because structural brain anomalies are reported in a subset of carriers, brain MRI should be considered when clinically indicated, for example, in the presence of seizures, abnormal head growth, focal neurological signs, or developmental concerns, to inform prognosis and surveillance.

For clinical practice, the implication is that all carriers warrant developmental surveillance, even when early milestones are reassuring. Furthermore, psychiatric risk may not manifest until adolescence or adulthood, meaning that surveillance must extend well beyond paediatric follow-up.

Neuroimaging studies provide additional context, with hippocampal malformations, corpus callosum hypoplasia, and ventricular dilatation frequently reported. These findings help explain the clinical variability but do not allow prognostic certainty. Families benefit when clinicians acknowledge both the elevated risk and the limits of prediction, and when they integrate developmental and psychiatric assessment into routine follow-up plans.

### 3.5. Hepatic Features

Hepatic involvement is less frequent but recognised. A proportion of carriers exhibit elevated serum transaminases, occasionally with hepatomegaly or imaging abnormalities. The underlying mechanism likely reflects the role of HNF1B in hepatocyte differentiation and metabolic regulation [3]. Most patients remain asymptomatic with preserved synthetic function, though a small subset develop chronic liver disease.

### 3.6. Reproductive and Genitourinary Features

Developmental expression of HNF1B and LHX1 in the urogenital tract explains the reproductive anomalies observed in some carriers. In females, Müllerian malformations such as a bicornuate uterus, uterine hypoplasia, or vaginal agenesis are reported [38,39]. In males, cryptorchidism, hypospadias, and infertility have been described. These features are often unrecognised until adolescence or adulthood.

### 3.7. Other Features

Additional systemic manifestations include ocular anomalies (colobomas, refractive errors, structural malformations) and, rarely, cardiovascular malformations [40]. Joint hypermobility and scoliosis have also been reported in a minority of cases. Epilepsy has been reported in individual cases, often associated with structural brain anomalies [35,41].

### 3.8. The 17q12 Recurrent Duplication Syndrome

The reciprocal duplication of the 1.4–1.5 Mb 17q12 interval causes a clinically distinct neurodevelopmental syndrome, providing an instructive contrast to the deletion phenotype. While both CNVs are associated with an increased risk for autism spectrum disorder, intellectual disability, and developmental delay, key differences exist. While both CNVs increase neurodevelopmental risk, deletions show stronger associations with ASD/ADHD and a trend toward schizophrenia; duplications often present with milder overall burden in non-index relatives. Formal comparative schizophrenia-risk estimates for duplication remain limited. Importantly, the duplication is less strongly associated with the characteristic renal structural anomalies or MODY5 seen in the deletion; when renal features occur in duplication carriers, they are typically milder. Large cohort analyses support this distinction, showing that duplication carriers are frequently identified in neurodevelopmental or psychiatric settings rather than through renal or metabolic disease. Learning difficulties, delayed language development, behavioural dysregulation, epilepsy, and occasionally structural brain differences all occur, though with broad variability. By contrast, deletion carriers follow a more predictable multisystem pattern involving renal and pancreatic vulnerability alongside neurodevelopmental involvement.

This phenotypic divergence underscores that the deletion’s multisystem features are not merely the inverse of duplication, but instead reflect the heightened sensitivity of renal, pancreatic, and hepatic tissues to reduced dosage of HNF1B and other genes in the interval. Increased dosage appears less disruptive to organ development yet may influence neurodevelopmental circuits, accounting for the predominance of behavioural and cognitive presentations in duplication carriers. This contrast also provides context for our cohort, whose clinical course aligns with the deletion-associated pattern described in the literature.

### 3.9. Diagnostic Strategies and Differential Diagnosis

Chromosomal microarray (CMA) remains the diagnostic gold standard, reliably identifying heterozygous deletions across 17q12. Parental testing subsequently confirmed the de novo origin, which was crucial for recurrence risk counselling.

When CMA is negative but suspicion remains high, HNF1B sequencing may reveal pathogenic variants, although these generally produce a narrower phenotype without the full neurodevelopmental spectrum [42,43]. The differential diagnosis is broad and includes autosomal dominant polycystic kidney disease, tuberous sclerosis complex, and PAX2-related renal-coloboma syndrome. Without genetic testing, phenotypic overlap may lead to misdiagnosis.

## 4. Discussion

**Figure 2:**
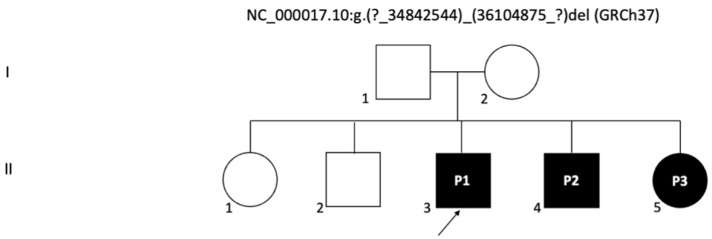
Pedigree chart of the family with a 17q12 deletion: The chart illustrates the inheritance pattern in the family, with the probands (P1, P2, and P3) indicated as affected by the 17q12 deletion, which includes the HNF1B gene. Circles represent females and squares represent males. Filled symbols indicate individuals affected by the genetic deletion. The arrow points to the first proband (P1).

The 17q12 recurrent deletion syndrome exemplifies the challenges of translating a well-defined genetic lesion into reliable clinical prognostication. It is striking that a uniform structural variant, encompassing the same 1.4 to 1.5 megabase interval in every carrier, can result in such diverse outcomes, ranging from asymptomatic adults to children with severe multi-organ disease or foetuses with lethal renal agenesis. The three siblings from a single family, all with the same inherited deletion, nevertheless demonstrated divergent renal, neurodevelopmental, and cognitive outcomes (Figure 2, Table 1).

Table 1 presents a structured overview of the three siblings with 17q12 deletion followed in our clinic over several years, with final assessments at 8, 5 and 4 years of age. The table integrates prenatal information, renal and neurodevelopmental findings, ophthalmological involvement, metabolic features and longitudinal follow up. Although all three children carry the same deletion, their phenotypes differ markedly. Sibling 1 shows complex renal malformations, ventriculomegaly and significant developmental delay, while sibling 2 has a milder course with transient metabolic abnormalities and normal renal imaging at the last review. Sibling 3 is distinguished by congenital ocular anomalies and mild neurodevelopmental impairment alongside early renal abnormalities. This harmonised format highlights both shared and divergent features across organ systems and clarifies the evolution of each child’s clinical profile over time.

### 4.1. Variable Expressivity and Prognostic Uncertainty

Our sibling series vividly illustrates high variability (Table 1). For families, this translates into a peculiar burden: the certainty of a deletion, but the uncertainty of what that deletion will mean in practice. Genetic counselling, therefore, cannot offer deterministic answers but must instead help families navigate a spectrum of plausible futures.

### 4.2. Renal Prognosis as a Model of Uncertainty

Renal involvement is the most frequent and recognisable manifestation of the deletion, and it provides a paradigmatic example of how structure and function diverge. Our three siblings vividly illustrate the breadth of renal involvement associated with the deletion. The eldest showed marked congenital abnormalities, with a multicystic right kidney evident from the prenatal period. Serial ultrasound demonstrated progressive atrophy of the right kidney until it became no longer visible, leaving an empty perirenal space, while the left kidney underwent clear compensatory hypertrophy. The renal parenchyma remained diffusely hyperechoic with absent corticomedullary differentiation, and small cortical cysts appeared in both kidneys. Additional urinary tract anomalies included a dysmorphic septate gallbladder, a bladder diverticulum and distal ureteral ectasia, all of which contributed to the complexity of the renal picture. Despite these abnormalities, renal function declined only gradually and stabilised with careful monitoring.

The second sibling had a milder presentation. Early scans showed bilateral multicystic involvement, with the left kidney more affected, although renal function was preserved. Mild proteinuria and phosphaturia appeared intermittently but resolved without specific intervention. By the age of five years, renal imaging had normalised, and serum creatinine and electrolytes remained within expected limits.

**Figure 3:**
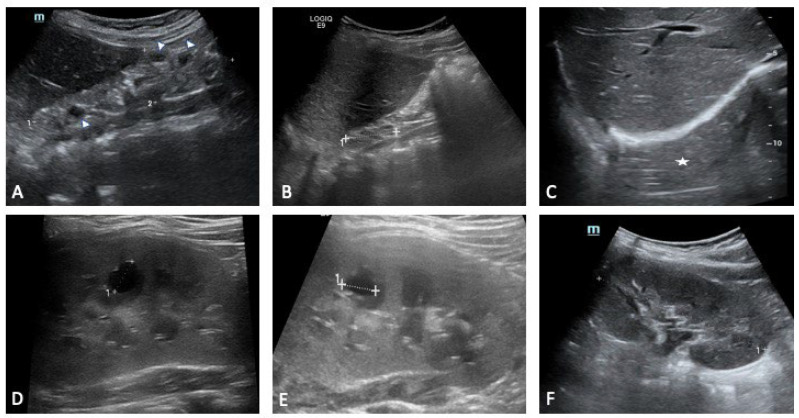
Sibling 1. Abdominal ultrasound examinations were performed at 3 (**A**,**D**), 4 (**B**,**E**), and 7 (**C**,**F**) years of age. (**A**–**C**) show progressive right kidney atrophy [maximal pole-to-pole diameter approximately 49 mm (dotted line 1 in (**A**)) and 33 mm (dotted line 1 in (**B**)); in (**C**), the right kidney is not clearly identifiable at the ultrasound exam («empty perirenal space», white asterisk)]. Renal parenchyma structure is altered, diffusely hyperechoic, with no clear differentiation between cortex and medulla (**A**,**B**). Some small cystic lesions can also be noted (arrowheads in (**A**)). Dotted line 2 in (**A**): antero-posterior diameter (20 mm). (**D**–**F**) show progressive compensatory hypertrophy of the left kidney [maximal pole-to-pole diameter approximately of 78 mm, 85 mm and 93 mm, respectively (the latter shown as dotted line 1 in (**F**))]. Some cystic lesions are identified in the renal parenchyma, with the most prominent of around 10 mm located in the middle third (dotted lines 1 in (**D**,**E**)).

**Figure 4:**
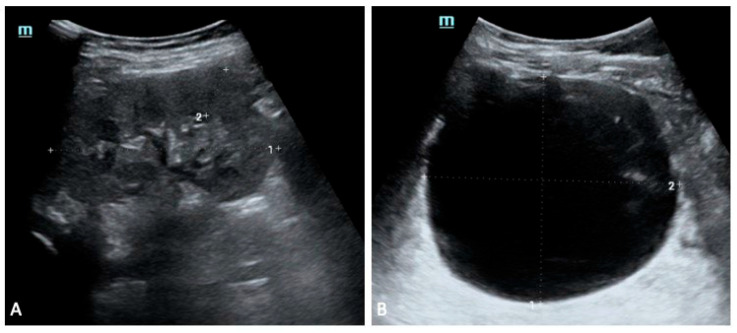
Sibling 2. Abdominal ultrasound exam performed at 5 years of age; images exploring the right (**A**) and left (**B**) perirenal spaces of the abdominal cavity. (**A**) shows a normal-sized kidney [pole-to-pole diameter approximately 79 mm (dotted line 1), anterior parenchymal thickness of 17 mm (dotted line 2)], with normal echogenicity. On the other hand, the left kidney structure is diffusely altered with several cysts seen within, ranging in size from a few millimeters to some centimeters (the most prominent one approximately measures 81 mm × 91 mm in the sagittal plane (dotted lines 1 and 2 in (**B**)).

**Figure 5:**
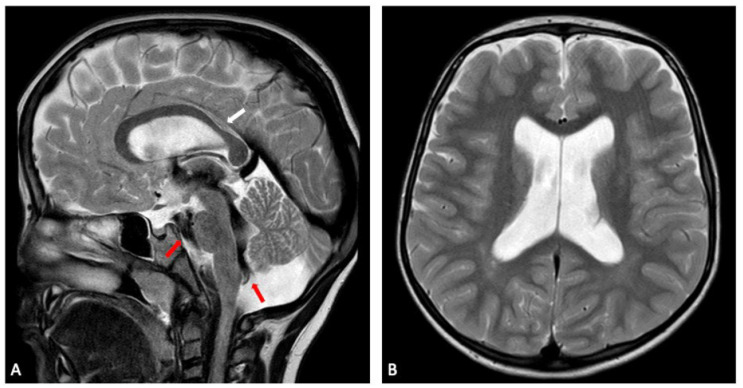
Sibling 1. Brain MRI; sagittal (**A**) and axial (**B**) T2-weighted turbo spin echo (TSE) images showing slight enlargement of the ventricular system. In (**A**), a prominent flow void (red arrows) is seen within the third ventricle, the cerebral aqueduct, the fourth ventricle and the prepontine cistern, expressing turbulent CSF flow, in keeping with no evident obstruction of CSF flow through ventricles and cisterns. Focal thinning of the posterior corpus callosum at the body-splenium junction can also be noted (white arrow in (**A**)).

The youngest sibling had prenatal signs of right kidney hyperechogenicity and bilateral pyelectasia. These changes improved over time, and renal function remained stable across follow up. Occasional proteinuria and phosphaturia were noted in early life, although subsequent biochemical tests showed steadier values. Compared with the older sibling, the renal course was relatively uncomplicated, despite the early anatomical findings (Figure 3, Figure 4 and Figure 5).

### 4.3. Endocrine and Metabolic Timing

In our series, the endocrine and metabolic profile varied across the three siblings, with some abnormalities emerging early and others remaining stable throughout follow up. The eldest sibling showed occasional mild hypercalcaemia during early childhood, a finding that settled with time and did not associate with renal impairment or disturbances in parathyroid hormone or vitamin D. The second sibling exhibited transient proteinuria and phosphaturia, although serum creatinine, glucose and electrolytes remained within reference ranges, and no persistent metabolic imbalance was documented. The youngest sibling had a more distinctive pattern, with episodes of hypercalcaemia accompanied by normal parathyroid hormone and vitamin D levels, together with sporadic proteinuria and phosphaturia. These episodes resolved spontaneously, yet they emphasise that metabolic disturbances can appear even when renal function seems relatively preserved.

None of the children had developed diabetes, impaired fasting glucose or other endocrine disorders at the time of their most recent evaluations. Their cases illustrate that normal findings in early childhood do not preclude later onset of metabolic features, especially as the risk of diabetes and hypomagnesaemia increases with age in individuals with this deletion. For this reason, continued surveillance remains essential, with periodic assessment.

### 4.4. Neurodevelopmental and Psychiatric Risk

Recognition of the neurodevelopmental and psychiatric consequences of the deletion has arguably been the most significant conceptual shift in recent years. Once regarded primarily as a renal and endocrine disorder, it is now acknowledged as one of the most penetrant copy number variants associated with autism spectrum disorder and schizophrenia. Large-scale genetic studies have quantified these risks, showing that carriers have a several-fold increased probability of such conditions compared with the general population. Attention deficit hyperactivity disorder, intellectual disability, epilepsy, and developmental delays are also prominent features in many carriers.

**Figure 6:**
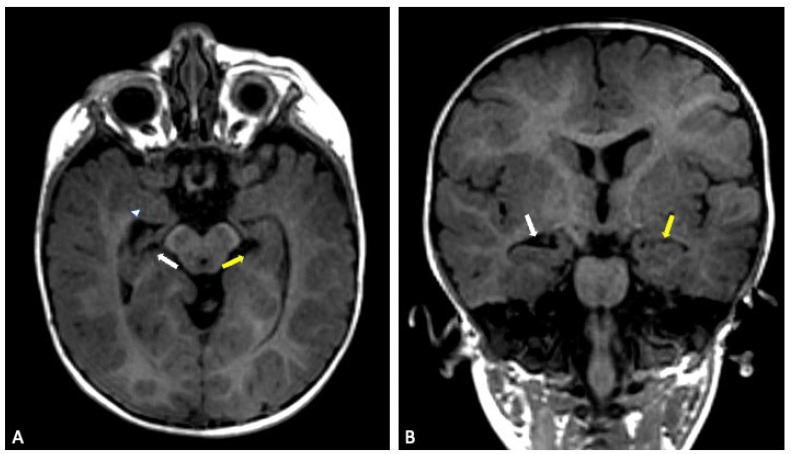
Sibling 3. Brain MRI; axial (**A**) and coronal (**B**) T1-weighted gradient echo (GE) images showing a reduced-size right hippocampus (white arrows), compared to left normal hippocampus (yellow arrows). On the right side, the compensatory enlargement of the temporal horn of the lateral ventricle can also be noted (arrowhead).

Our sibling series exemplifies this variability (Figure 5 and Figure 6). The eldest child presented with early hypotonia, delayed motor and cognitive development and increasing macrocephaly during infancy. Brain MRI revealed enlargement of the ventricular system, thinning of the posterior corpus callosum and clear hippocampal atrophy. Additional findings included subtle cerebellar hemisphere hypoplasia and a cerebrovascular variant involving asymmetric fusion of the caudal basilar artery together with ectasia of a posterior thalamic perforating branch. These structural abnormalities corresponded to persistent executive and attentional difficulties that remained evident at school age.

The second sibling displayed a milder pattern. Developmental progress was delayed but more evenly paced, and febrile seizures occurred in early childhood. Neuroimaging identified a slight incision of the corpus callosum isthmus, mild pons hypoplasia and a reduction in hippocampal volume bilaterally. Mild hypotonia was also noted during infancy, although cognitive outcomes remained within a broader low average range. Compared with the eldest sibling, functional difficulties were less pronounced, and day to day adaptive skills improved steadily across follow up.

The youngest sibling showed the subtler clinical profile despite having structural abnormalities on imaging. MRI demonstrated a reduced right hippocampal volume with compensatory enlargement of the temporal horn, alongside thinning of the corpus callosum and mild pons hypoplasia. Motor and language skills developed more slowly than expected, but the overall course was steadier, and no seizures were reported. This combination of anatomical findings and comparatively mild clinical expression is in keeping with observations from larger cohorts, where even siblings carrying the same deletion can differ greatly in their developmental and behavioural outcomes.

### 4.5. Other Features

In our series, ophthalmological involvement was confined to the youngest sibling, who displayed congenital bilateral vascularised corneal leukoma, microphthalmia, exotropia and nystagmus, together with subtle irregularities of the optic nerve papillae. These findings illustrate that ocular anomalies can be prominent even when other systemic features are comparatively mild. The other two siblings had normal ophthalmological examinations, emphasising the variable extent to which visual pathways may be affected in individuals with the deletion.

### 4.6. Counselling and Ethics in Prenatal and Paediatric Settings

The ethical challenges posed by the 17q12 deletion stem directly from its variability. Prenatal detection exemplifies this problem. Ultrasonographic findings of echogenic or cystic kidneys may prompt genetic testing and confirmation of the deletion, yet the postnatal outcomes range from normal renal function to severe disease. In some cases the deletion is detected incidentally in asymptomatic parental carriers, adding further complexity to counselling.

Counselling in this context requires careful explanation that penetrance is incomplete and expression unpredictable, even within families. Families often want to know whether their child will develop diabetes, whether renal function will decline, or whether psychiatric illness will emerge. The only honest answer is that risk is increased but timing and severity are unpredictable.

### 4.7. Management as a Life-Course Framework

Given the multisystem and evolving nature of the syndrome, management is best conceptualised as a life-course framework rather than as an organ-based checklist. Four inflection points can be recognised. The first is diagnosis, often triggered by prenatal or early postnatal imaging. At this stage families require a clear explanation of the deletion, a written plan of surveillance across organ systems, and contact points for genetics and subspecialists. The second is early childhood, when development is most plastic. Here the priority is to identify speech, language, motor, or social delays early and to provide timely intervention, while maintaining regular checks of renal function and biochemical markers. The third is adolescence, when metabolic risk increases and psychiatric disorders often emerge. Annual glucose assessment, targeted endocrine review, and routine mental health screening embedded within clinic visits are pragmatic strategies. The fourth is transition to adult care, which should be prepared in advance with explicit handover of renal, endocrine, and neurodevelopmental histories and surveillance plans.

This life-course approach addresses the central challenge of variability by acknowledging that different features emerge at different times. It also reduces fragmentation of care by clarifying responsibilities across specialties and across the transition from paediatrics to adult medicine. The surveillance timetable (Table 2) carries weight beyond its practical use, because it offers a way to bring order to a condition that can otherwise feel unpredictable. It shows how clinical priorities shift as children grow, and it helps families understand why regular review remains important even when early findings appear reassuring. Renal, metabolic and neurodevelopmental features do not emerge at the same pace, and this uneven progression is exactly why a lifelong plan is needed. By emphasising monitoring across the entire life course, the discussion highlights that good management depends as much on steady observation as on individual test results. Families often say that having a clear sense of what will be checked, and at which stage, makes the uncertainty easier to navigate. A focus on lifetime surveillance therefore strengthens clinical care and supports families who must plan for the long term despite the variability of the syndrome.

### 4.8. Pragmatic Surveillance Pathway and Transition Planning

In practical terms, surveillance is most effective when it is described to families as a timetable rather than a list of potential complications. In infancy and early childhood, the emphasis is on establishing a renal and developmental baseline [44]. Renal ultrasonography, serum creatinine with estimated glomerular filtration, urinalysis for protein and blood, and blood pressure measurement provide a functional picture that complements structural imaging; repeating these at clinically sensible intervals allows the team to detect quiet changes before symptoms emerge. Developmental reviews embedded in routine clinics, with low thresholds for referral to speech and language therapy, physiotherapy or neuropsychology, make use of the window of developmental plasticity in the early years. Where electrolyte profiles are normal at presentation, families should understand that periodic checks of glucose, magnesium and urate are anticipatory rather than reactive and that stability is a common, acceptable outcome.

As children approach later childhood, the cadence shifts to reflect age-dependent risks. Annual assessment of fasting glucose or glucose tolerance, combined with continued monitoring of renal function, proteinuria and blood pressure, aligns clinical priorities with the natural history seen across cohorts. Simple screening questions about attention, learning, mood and sleep can be integrated into clinic conversations without adding procedural burden, and they normalise the expectation that neurodevelopment evolves across childhood rather than being fixed at diagnosis. When abnormalities arise, early intervention reduces downstream complexity and improves family confidence in the follow-up plan [44,45].

Transition to adult services merits explicit preparation. A concise handover document that summarises renal imaging history, functional trends, episodes of hypertension or proteinuria, biochemical trajectories including glucose and magnesium, and any neurodevelopmental or educational supports prevents informational loss at the boundary of care. Reproductive counselling should be offered in late adolescence, with attention to the recognised spectrum of Müllerian anomalies in females and cryptorchidism or hypospadias in males, and with a reminder that parental phenotype does not predict expression in offspring even when the deletion is inherited. Finally, cascade testing within families, undertaken with sensitive counselling about incomplete penetrance, can identify at-risk relatives who may benefit from proportionate surveillance despite minimal or absent symptoms.

### 4.9. Future Directions

Despite substantial progress, important gaps remain. The natural history of the syndrome is still incompletely characterised, particularly with respect to long-term renal function, timing of diabetes onset, and psychiatric risk. Cross-sectional cohorts, while informative, cannot answer these questions. Longitudinal registries that combine detailed phenotyping with genetic data are urgently needed. Functional studies of genes within the deleted interval beyond HNF1B, such as LHX1, SYNRG, and ZNHIT3, may clarify their contributions to neurodevelopmental and psychiatric phenotypes. Animal models provide partial insight but cannot capture the full spectrum of human psychiatric outcomes.

Another area requiring attention is the development of ethical frameworks for prenatal counselling. Current practice is hampered by the inability to predict severity, and families often struggle with decisions in the absence of robust prognostic markers. Probabilistic counselling that explicitly incorporates uncertainty, supported by written resources and structured decision aids, may improve this process. Finally, international collaboration is essential. The rarity of the syndrome means that no single centre can accumulate sufficient cases for definitive studies. Coordinated registries and shared protocols would not only enhance research but also support the development of evidence-based surveillance guidelines.

### 4.10. Genetic Counselling

Genetic counselling is a cornerstone of management for 17q12 recurrent deletion syndrome, given its autosomal dominant inheritance, variable expressivity, and incomplete penetrance for specific features.

#### 4.10.1. Mode of Inheritance and Recurrence Risk

Approximately 70–75% of probands have a de novo deletion, while 25–30% have inherited it from a parent. The recurrence risk for parents of a proband with a de novo deletion is low (<1%) but greater than the general population due to the theoretical possibility of germline mosaicism. For offspring of an affected individual, the risk of inheritance is 50%.

#### 4.10.2. Evaluation of Relatives and Cascade Testing

Once the deletion is identified in a proband, cascade testing of parents is essential to determine recurrence risk and identify often overlooked, mildly affected relatives. If a parent is found to carry the deletion, offering genetic counselling and testing to the parents’ at-risk siblings should be considered.

#### 4.10.3. Prenatal Testing and Preimplantation Genetic Testing (PGT)

For families where a parent carries the deletion, prenatal testing is available via chorionic villus sampling (CVS) or amniocentesis. Preimplantation genetic testing (PGT) may also be an option. Counselling in these contexts is complex due to the profound variability and the inability to predict phenotype. The identification of echogenic kidneys on prenatal ultrasound should prompt consideration of CMA, and if the deletion is found, families must be counselled about the wide spectrum of possible outcomes, from isolated benign renal findings to severe multi-system involvement.

### 4.11. De Novo-Germline Mosaicism

The term de novo indicates that the deletion is not detectable in the parents’ blood samples and therefore appears to have arisen for the first time in the child. It does not mean that the event occurred at birth or during childhood, but rather that it was not inherited through the usual parental line. Importantly, a de novo result does not completely exclude the possibility of recurrence, because one parent may carry the deletion only in a proportion of germ cells while having a normal blood test. This situation, known as germline mosaicism, cannot be ruled out by standard parental testing and creates a small but real risk of the same alteration occurring again in future pregnancies. In such cases, a parent may carry the deletion in a proportion of germ cells while having a normal blood result, creating a small but meaningful recurrence risk in future pregnancies. This possibility is well recognised in chromosomal microdeletion syndromes and should be discussed during counselling, particularly when parents wish to understand residual reproductive risk despite negative testing.

## 5. Conclusions

The 17q12 recurrent deletion syndrome is a paradigmatic paediatric genomic disorder that combines renal and metabolic risk with a significant neurodevelopmental and psychiatric burden and a high degree of variability between and within families. Early genetic diagnosis enables anticipatory care and tailored counselling, yet prognosis cannot be inferred from genotype or baseline imaging alone. The addition of structured genetic counselling and a standardised, life-course surveillance plan provides a robust framework for managing this complex and variable condition. Until longitudinal data permit better prediction, clinical management should remain multidisciplinary, life-course oriented and flexible, with surveillance that is scaled to the individual and explained clearly to families. Finally, clinicians should be aware that recurrent microdeletions and microduplications in this region may also arise from cryptic inversions in one of the parents. This mechanism increases the recurrence risk beyond that expected for a truly de novo event and cannot be detected by standard chromosomal microarray or sequencing; targeted three-colour FISH probe sets can reveal such inversions when they are large enough to be resolved cytogenetically [46].

## Figures and Tables

**Table 1:** Clinical summary.

Parameter	Sibling 1 (M, Followed to 8 Years)	Sibling 2 (M, Followed to 5 Years)	Sibling 3 (F, Followed to 4 Years)
Age of onset	Prenatal	6 months	Birth
Prenatal findings	Multicystic right kidney	Unremarkable prenatal course	Right kidney hyperechogenicity and bilateral pyelectasia
Renal features	Bilateral multicystic kidney disease; right kidney nonfunctional; dysmorphic septate gallbladder; bladder diverticulum with distal ureteral ectasia; occasional mild hypercalcaemia	Bilateral multicystic kidney disease, more severe on the left; mild transient proteinuria; phosphaturia; normal creatinine and electrolytes; normal ultrasound at 5 years	Right renal hyperechogenicity; bilateral pyelectasia; sporadic proteinuria; phosphaturia; occasional hypercalcaemia
Neurological features	Delayed motor and cognitive milestones; macrocephaly; neonatal hypotonia; attention difficulties	Macrocephaly; cognitive and motor delays; febrile seizures; mild hypotonia	Slight neurodevelopmental impairment; delayed motor milestones
MRI findings	Enlarged supra and infratentorial ventricles; corpus callosum thinning; cerebellar hemisphere hypoplasia; hippocampal atrophy; cerebrovascular variant involving the basilar artery and posterior thalamic perforators	Slight incision of corpus callosum isthmus; mild pons hypoplasia; bilateral hippocampal volume reduction	Right hippocampal reduction; corpus callosum anomalies; mild pons hypoplasia
Ophthalmological features	Normal examination	Normal examination	Congenital bilateral vascularised corneal leukoma; microphthalmia; exotropia; nystagmus; optic nerve papilla irregularities
Metabolic features	Occasional mild hypercalcaemia	Transient proteinuria and phosphaturia with normal renal function	Hypercalcaemia with normal parathyroid hormone and vitamin D; sporadic proteinuria and phosphaturia
Follow up findings (last evaluation)	Stable renal function; persistent learning and attention difficulties; ongoing developmental support through to 8 years	Normal renal imaging and stable biochemical profile through to 5 years; regular developmental monitoring	Stable renal function; ongoing ophthalmology and developmental care through to 4 years
Genetic findings	17q12 deletion	17q12 deletion	17q12 deletion

**Table 2:** Proposed Surveillance Schedule for 17q12 Recurrent Deletion Syndrome.

System	Infancy & Early Childhood (0–5 Years)	Childhood & Adolescence (6–18 Years)	Adulthood (18+ Years)
Renal	Renal US at Dx; BP; serum Cr/eGFR; urinalysis 12 months after diagnosis	BP, serum Cr/eGFR, urinalysis, every 2–3 years. Repeat renal US if clinical change.	BP, serum Cr/eGFR, urinalysis every 3–5 years. Monitor for hypertension & proteinuria.
Metabolic/Endocrine	Baseline glucose, Mg, urate, LFTs.	Annual fasting glucose or HbA1c; Mg, urate, LFTs annually.	Annual annual HbA1c. Monitor Mg, urate.
Neurodevelopmental	Formal developmental assessment at Dx. Annual developmental screening.	Annual assessment of educational progress, behavior, and mental health.	Monitor for late-onset psychiatric conditions (e.g., schizophrenia, anxiety).
Other	Baseline brain MRI considered. Ophthalmology evaluation if indicated.	Reproductive counseling in late adolescence. hearing screening. annual ophthalmology check.	Ongoing reproductive and genetic counseling.

Inflexion Points in Care. • Diagnosis: Provide a clear explanation, a written surveillance plan (like Table 1), and a genetics contact. • Early Childhood: Prioritise early identification of developmental delays and provide timely interventions. • Adolescence: Increase vigilance for metabolic issues (diabetes onset) and emergent psychiatric symptoms. Initiate reproductive counselling. • Transition to Adult Care: A concise handover document summarising the patient’s history and future surveillance needs is critical to prevent care fragmentation.

## Data Availability

No new data were created or analysed in this study.

## Abbreviations

The following abbreviations are used in this manuscript:

CAKUT	Congenital anomalies of the kidney and urinary tract
MODY5	Maturity-onset diabetes of the young type 5
ESKD	End-stage kidney disease
CMA	Chromosomal microarray analysis
CNV	Copy number variant
MRI	Magnetic resonance imaging
US	Ultrasound
eGFR	Estimated glomerular filtration rate
LFTs	Liver function tests
